# Evaluation of the learning curve for conformal sphincter preservation operation in the treatment of ultralow rectal cancer

**DOI:** 10.1186/s12957-022-02541-1

**Published:** 2022-03-30

**Authors:** Hai-bo Ding, Lin-hui Wang, Ge Sun, Guan-yu Yu, Xian-hua Gao, Kuo Zheng, Hai-feng Gong, Jin-ke Sui, Xiao-ming Zhu, Wei Zhang

**Affiliations:** 1 Department of Colorectal Surgery, Changhai Hospital, Naval Medical University, Shanghai, 200433 China; 2 College of Education, Zhejiang University, Zhejiang, 310058 Hangzhou China; 3 University Medical Center of Groningen, 9713GZ Groningen, Netherlands

**Keywords:** Learning curve, Ultralow rectal cancer, Conformal sphincter preservation operation, Operation time

## Abstract

**Background:**

To investigate the learning curve of conformal sphincter preservation operation (CSPO) in the treatment of ultralow rectal cancer and to further explore the influencing factors of operation time.

**Methods:**

From August 2011 to April 2020, 108 consecutive patients with ultralow rectal cancer underwent CSPO by the same surgeon in the Department of Colorectal Surgery of Changhai Hospital. The moving average and cumulative sum control chart (CUSUM) curve were used to analyze the learning curve. The preoperative clinical baseline data, postoperative pathological data, postoperative complications, and survival data were compared before and after the completion of learning curve. The influencing factors of CSPO operation time were analyzed by univariate and multivariate analysis.

**Results:**

According to the results of moving average and CUSUM method, CSPO learning curve was divided into learning period (1–45 cases) and learning completion period (46–108 cases). There was no significant difference in preoperative clinical baseline data, postoperative pathological data, postoperative complications, and survival data between the two stages. Compared with the learning period, the operation time (*P* < 0.05), blood loss (*P* < 0.05), postoperative flatus and defecation time (*P* < 0.05), liquid diet time (*P* < 0.05), and postoperative hospital stay (*P* < 0.05) in the learning completion period were significantly reduced, and the difference was statistically significant. Univariate and multivariate analysis showed that distance of tumor from anal verge (≥ 4cm vs. < 4cm, *P* = 0.039) and T stage (T3 vs. T1-2, *P* = 0.022) was independent risk factors for prolonging the operation time of CSPO.

**Conclusions:**

For surgeons with laparoscopic surgery experience, about 45 cases of CSPO are needed to cross the learning curve. At the initial stage of CSPO, beginners are recommended to select patients with ultralow rectal cancer whose distance of tumor from anal verge is less than 4 cm and tumor stage is less than T3 for practice, which can enable beginners to reduce the operation time, accumulate experience, build self-confidence, and shorten the learning curve on the premise of safety.

## Background

Miles operation [1] is considered as the standard treatment for ultralow rectal cancer with the lower edge of the tumor less than 2 cm from the dentate line. However, the stomal complications [2] and long-term nursing of permanent colostomy have seriously reduced the quality of life [3], role, and social function [4] of patients after Miles. Hence, many patients with ultralow rectal cancer refuse colostomy before operation [5]. In recent years, preoperative neoadjuvant chemoradiotherapy (nCRT) [6, 7] and laparoscopic technology have developed rapidly. People also have a deeper understanding of the safety of rectal cancer with a distance of less than 2 cm from the distal resection margin [8]. The maturity of technique and anatomic basis of anal-preserving operation not only improves its oncological prognosis [9] and functional prognosis [10] but also promotes the expansion of its related research and application.

Considering the anatomic characteristics of the anal canal, the Department of Colorectal Surgery, Shanghai, Changhai Hospital, proposed the CSPO [11–13] for ultralow rectal cancer. As a new anal-preserving operation, it combined with total mesorectal excision (TME) [14, 15], coloanal anastomosis [16], eversion pull-through resection [17], anal canal dissection, local tumor resection [18], natural orifice translumenal endoscopic surgery (NOTES) [19], and so forth. Since August 2011, our group has successfully passed the CSPO learning curve and achieved satisfactory results. By April 2020, we have completed 123 cases of CSPO. The favorable oncology and functional prognosis of patients with CSPO have gradually been confirmed by relevant studies in our center, and relevant literature has also been published [11–13].

As a new anal-preserving operation, CSPO has deep experience requirements for laparoscopic surgery, perianal disease surgery, and manual coloanal anastomosis. To ensure the safety of CSPO, it is important to evaluate its learning curve and establish an appropriate training program. However, the learning curve of CSPO is not clear at present. In this study, we evaluated the learning curve by analyzing the clinical data of 108 consecutive patients who underwent laparoscopic CSPO by a same surgeon. By moving average, CUSUM, we systematically evaluated the learning curve of CSPO and further analyzed the influencing factors of CSPO operation time.

## Methods

### Data collection and study design

We retrieved the clinical data of 123 consecutive ultralow rectal cancer patients, who were successively implemented CSPO by the same surgeon in the colorectal surgery department of Shanghai Changhai Hospital from August 2011 to April 2020. The surgeon had experience of performing more than 200 cases of routine laparoscopic surgery for colorectal cancer over 10 years. All patients met the following criteria: (1) proven well-differentiated rectal adenocarcinoma with digital rectal examination, colonoscopy, and biopsy, and distant metastasis was excluded by MRI; (2) the tumor did not infiltrate the intersphincteric space; (3) good anal function before the operation; (4) the distance from the inferior tumor edge to the dentate line was less than 2 cm; (5) the diameter of the tumor was less than 3 cm and occupied less than 1/3 circumference of the lumen (for patients with limited canceration and shallow infiltration, the indication of mass diameter is appropriately relaxed); (6) neoadjuvant therapy in the case of preoperative stage T3-T4 or N+ or if the circumferential resection margin (CRM) was considered positive; and (7) American Society of Anesthesiologists (ASA) class ≤ 3. All patients who met the inclusion criteria after neoadjuvant therapy were treated with CSPO 6–8 weeks after the end of treatment.

The operation time is the main index to evaluate the learning curve, and it is also the main outcome of this study. In addition to CSPO, 15 patients underwent other complicated simultaneous operations, including simultaneous hepatectomy, hysterectomy, adnexectomy, and other operations that significantly affect the operation time. Excluding these 15 cases, a total of 108 cases were included in this study. Preoperative clinical baseline data, surgical and postoperative pathological data, postoperative complications, and survival data were the secondary outcomes of this study.

The preoperative status of patients was evaluated by ASA classification. Postoperative complications were defined as complications occurred within 30 days after operation, which were scored according to Clavien–Dindo classification. Postoperative infection was defined as fever with body temperature higher than 38 °C and hemogram rising within 30 days after operation, which could be relieved after antibiotic therapy. The number of harvested lymph nodes was the number of lymph nodes detected by postoperative pathology. The nature and staging of the tumor were all postoperative staging. Therefore, some patients with pathological complete response (pCR) after nCRT were classified as non tumor/scar group, the degree of differentiation was classified as no mass/premalignant lesion group, and T stage was classified as T0 group. PFS3 is the progression-free survival rates at 3 years. OS3 is the overall survival rates at 3 years.

We analyzed the learning curve for CSPO using moving average and CUSUM curve. The moving average uses the average value of the data over a period of time to replace the single case data, which can eliminate the influence of data fluctuation and observe the long-term change trend of the data. CUSUM curve can be used to observe the small changes between a case data and the overall data, and the vertex is the turning point of the overall data. According to the cutoff value of the vertex of CUSUM curve, the CSPO learning curve can be divided into learning period and learning completion period. In order to explore the influencing factors of the operation time of CSPO and to provide the basis for beginners to select the appropriate cases, the operation time of CSPO was further divided into three groups according to the interquartile interval. The first 25% was included in group A, the middle 50% in group B, and the last 25% in group C. Group A and group C were compared with group B for univariate and multivariate analysis. The results of univariate analysis (*P* < 0.05) were included in multivariate analysis. The results of multivariate analysis (*P* < 0.05) were considered as independent risk factors affecting the operation time of CSPO (Tables 2 and 3).

This study was approved by the hospital ethics committee, and informed consent was obtained from patients and their families.

### Surgical techniques

Referring to the literature [11], CSPO can be divided into abdominal operation and perineal operation.

#### Abdominal operation

First, the sigmoid colon was freed, and the root of mesenteric artery was ligated. Then, according to the principle of TME, the rectum is freed to the level of hiatal ligament, the hiatal ligament is cut off, and the dissection is stopped at the entrance of sphincter space, which is one of the important differences between CSPO and intersphincteric resection (ISR). During the operation, the autonomic nerve was protected, and the intestinal canal was severed at the junction of rectum and sigmoid colon.

#### Perineal operation

First, the anus was enlarged to 3–4 finger widths, and the rectum was prolapsed through the anus. Then, the conformal resection line was designed according to the location and size of the tumor. The key to the operation is to preserve the rectal wall, dentate line, and internal sphincter on the opposite side of the tumor as much as possible on the premise of ensuring that the distal resection margin under direct vision is ≥ 1 cm. In addition, avoid separating the sphincter space to prevent damage of the nerve tissue in it and protect the function of the remaining sphincter as much as possible. In the case that the rectum can not be prolapsed through the anus, conformal resection of tumor can be performed by transanal first. Then the specimen was pulled out, and the rectal stump was sutured intermittently. Routine intraoperative frozen section diagnosis was performed to ensure the safety of the distal resection margin. After closing the rectal stump, a 25 mm tubular stapler (CDH25, Johnson & Johnson, USA) was placed through the anus for anastomosis. The anastomosis position was selected on the side with more rectum, and the dentate line and internal sphincter on the opposite side of the tumor were retained as much as possible. If the rectal stump is too short, manual anastomosis can also be used (first, one 3-0 absorbable thread was used for upper, lower, left, and right stitches and then two 3-0 barbed threads were used for continuous suture; each thread was sutured half a circle).

All patients underwent laparoscopic surgery and temporarily ileostomy, which were completed by the same surgeon and the same operation team.

### Statistical analysis

The statistical analysis of this study is based on SPSS 23.0 statistical software, the moving average curve is drawn by Excel, and the CUSUM curve is drawn and fitted by SPSS 23.0 statistical software. The counting data were expressed by the rate (%), and the normal distribution measurement data were expressed by means ± SD, and those data were all accurate to the last decimal point. *χ*^2^ test or Fisher exact probability method was used to compare the counting data between groups; *t*-test was used to compare the measurement data between the two groups. *P* < 0.05 was considered to indicate a statistically significant difference between the data sets.

## Results

### Baseline characteristics

The baseline characteristics are described in Table 1. Of 108 patients, 70 were male (64.8%) and 38 were female (35.2%) with a mean age of 57 years and mean BMI of 23 kg/m^2^. Thirty-seven patients (34.3%) underwent preoperative neoadjuvant therapy. The mean intraoperative blood loss was 168.8 ml. The mean number of harvested lymph nodes was 12.8. The mean maximum diameter of mass was 3 cm. The mean distal resection margin (DRM) was 0.7 cm, and all patients had R0 resection and negative CRM. There was no 30-day mortality. The overall postoperation complication rate was 16.7%, and the postoperative infection rate was 9.3%. According to the Clavien–Dindo classification, overall incidence of grade 2 or higher postoperative complications was 13.9% (15/108). The PFS3 were 88%. The OS3 were 93.5%.

**Table 1:** Baseline characteristics

	Total (*n* = 108)	Learning period (45 cases)	Completion period (63 cases)	*P*	
Gender (cases)				0.247	
Male	70 (64.8%)	32 (29.6%)	38 (35.2%)		
Female	38 (35.2%)	13 (12%)	25 (23.2%)		
Age (years)	57.0 ± 10.9	56.3 ± 12.0	57.5 ± 10.0	0.578	
BMI (Kg/m^2^)	23.0 ± 3.3	23.2 ± 3.7	22.9 ± 3.0	0.633	
ASA classification				0.278	
1	9 (8.3%)	5 (4.6%)	4 (3.7%)		
2	92 (85.2%)	38 (35.2%)	54 (50%)		
3	7 (6.5%)	2 (1.9%)	5 (4.6%)		
4	0	0	0		
Previous abdominal surgical history				0.535	
Yes	12 (11.1%)	4 (3.7%)	8 (7.4%)		
No	96 (88.9%)	41 (38%)	55 (50.9%)		
Neoadjuvant therapy				0.16	
Yes	37 (34.3%)	12 (11.1%)	25 (23.2%)		
No	71 (65.7%)	33 (30.5%)	38 (35.2%)		
CEA (ng/ml)				0.781	
Abnormal	23 (21.3%)	9 (8.3%)	14 (13%)		
Normal	85 (78.7%)	36 (33.3%)	49 (45.4%)		
CA19-9 (U/ml)				0.947	
Abnormal	7 (6.5%)	3 (2.8%)	4 (3.7%)		
Normal	101 (93.5%)	42 (38.9%)	59 (54.6%)		
Intraoperative blood loss (ml)	168.8 ± 87.2	204.7 ± 99.7	143.1 ± 67.1	0	
Number of harvested lymph nodes	12.8 ± 4.6	13.3 ± 4.5	12.4 ± 4.7	0.323	
Distance of tumor from anal verge (cm)	3.4 ± 1.1	3.2 ± 0.9	3.5 ± 0.9	0.192	
Distal resection margin (cm)	0.7 ± 0.5	0.6 ± 0.4	0.7 ± 0.6	0.393	
Maximum diameter of mass (cm)	3.0 ± 1.4	3.1 ± 1.7	2.9 ± 1.2	0.342	
Preoperative tumor gross type				0.683	
No/scar	10 (9.3%)	5 (4.6%)	5 (4.7%)		
Ulcerative	61 (56.5%)	23 (21.3%)	38 (35.2%)		
Proliferated	36 (33.3%)	16 (14.8%)	20 (18.5%)		
Infiltrated	1 (0.9%)	1 (0.9%)	0		
The degree of tumor differentiation				0.672	
No/premalignant lesion	12 (11.1%)	5 (4.6%)	7 (6.5%)		
High differentiation	6 (5.6%)	1 (0.9%)	5 (4.7%)		
Medium differentiation	83 (76.8%)	36 (33.3%)	47 (43.5%)		
Low differentiation	7 (6.5%)	3 (2.8%)	4 (3.7%)		
cT stage				0.132	
0	0	0	0		
1	18 (16.7%)	8 (7.4%)	10(9.3%)		
2	51 (47.2%)	24 (22.2%)	27 (25%)		
3	38 (35.2%)	13 (12%)	25 (23.2%)		
4	1 (0.9%)	0	1 (0.9%)		
cN stage				0.883	
0	63 (58.3%)	28 (25.9%)	35 (32.4%)		
1	33 (30.6%)	12 (11.1%)	21 (19.5%)		
2	12 (11.1%)	5 (4.6%)	7 (6.5%)		
pT stage				0.559	
0	6 (5.6%)	3 (2.8%)	3 (2.8%)		
1	22 (20.4%)	9 (8.3%)	13 (12.1%)		
2	52 (48.1%)	23 (21.3%)	29 (26.8%)		
3	28 (25.9%)	10 (9.3%)	18 (16.6%)		
4	0	0	0		
pN stage				0.448	
0	89 (82.4%)	35 (32.4%)	54 (50%)		
1	16 (14.8%)	9 (8.3%)	7 (6.5%)		
2	3 (2.8%)	1 (0.9%)	2 (1.9%)		
pTNM comprehensive staging				0.642	
0	6 (5.6%)	3 (2.8%)	3 (2.8%)		
I	66 (61.2%)	27 (25%)	39 (36.2%)		
II	18 (16.6%)	5 (4.6%)	13 (12%)		
III	18 (16.6%)	10 (9.3%)	8 (7.3%)		
IV	0	0	0		
Postoperative complications				0.432	
Yes	18 (16.7%)	9 (8.3%)	9 (8.4%)		
No	90 (83.3%)	36 (33.3%)	54 (50%)		
Postoperative infection				0.217	
Yes	10 (9.3%)	6 (5.6%)	4 (3.7%)		
No	98 (90.7%)	39 (36.1%)	59 (54.6%)		
Classification of complications				0.825	
0	90 (83.3%)	36 (33.3%)	54 (50%)		
I	3 (2.8%)	3 (2.8%)	0		
II	14 (13%)	6 (5.6%)	8 (7.3%)		
III	1 (0.9%)	0	1 (0.9%)		
IV	0	0	0		
V	0	0	0		
Flatus and defecation time (days)	2.1 ± 0.6	2.3 ± 0.4	2.1 ± 0.6	0	
Postoperative liquid diet time (days)	2.6 ± 0.6	2.7 ± 0.5	2.5 ± 0.7	0	
Postoperative hospital stay (days)	6.2 ± 2.6	6.9 ± 2.9	5.6 ± 2.2	0.002	
Closure of stoma				0.201	
Yes	102 (94.4%)	44 (40.7%)	58 (53.7%)		
No	6 (5.6%)	1 (0.9%)	5 (4.7%)		
Duration of stoma (median months)	8	7	9	0.044	
PFS3	95/108; 88.0%	38/45; 84.4%	57/63; 90.5%	0.342	
OS3	101/108; 93.5%	40/45; 88.9%	61/63; 96.8%	0	

Values are reported as mean ± SD or as median and interquartile range

CEA > 5 ng/ml and CA19-9 > 37 U/ml were judged as abnormal

### Learning curve

**Fig. 1:**
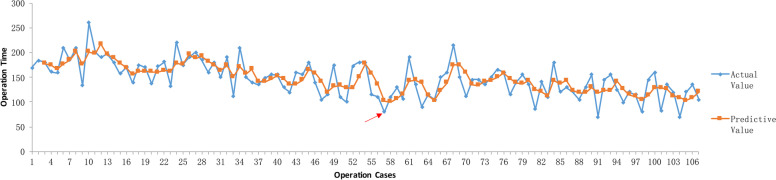
Moving average curve of CSPO operation time. →The first lowest point of the moving average curve

Based on the moving average curve of CSPO operation time (Fig. 1), it can be seen that with the accumulation of operation cases, the moving average is gradually decreasing, and the first lowest point appears at 57 cases. Although the curve fluctuated after that, it gradually stabilized. Combined with the lag effect of moving average, it can be predicted that there is a learning curve in CSPO, and the cutoff point value is before 57 cases.

**Fig. 2:**
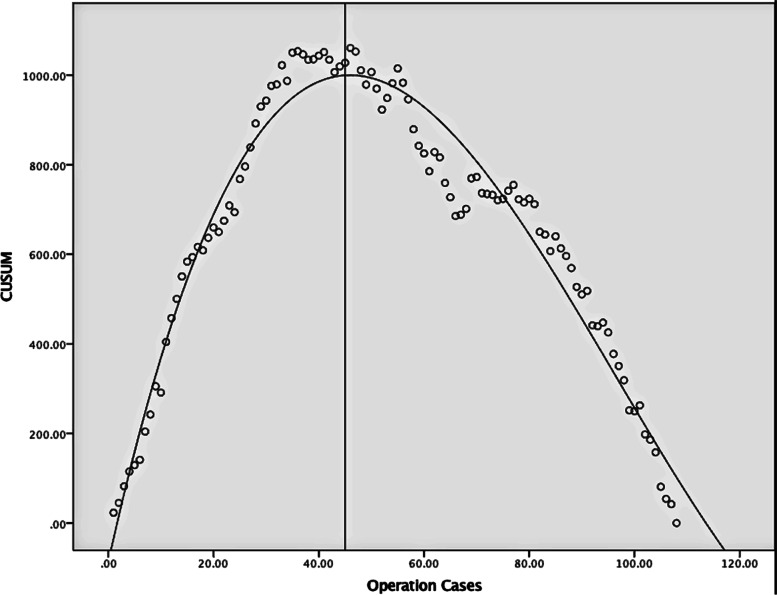
CUSUM curve of CSPO operation time. │Vertical line through CUSUM curve cutoff point

**Fig. 3:**
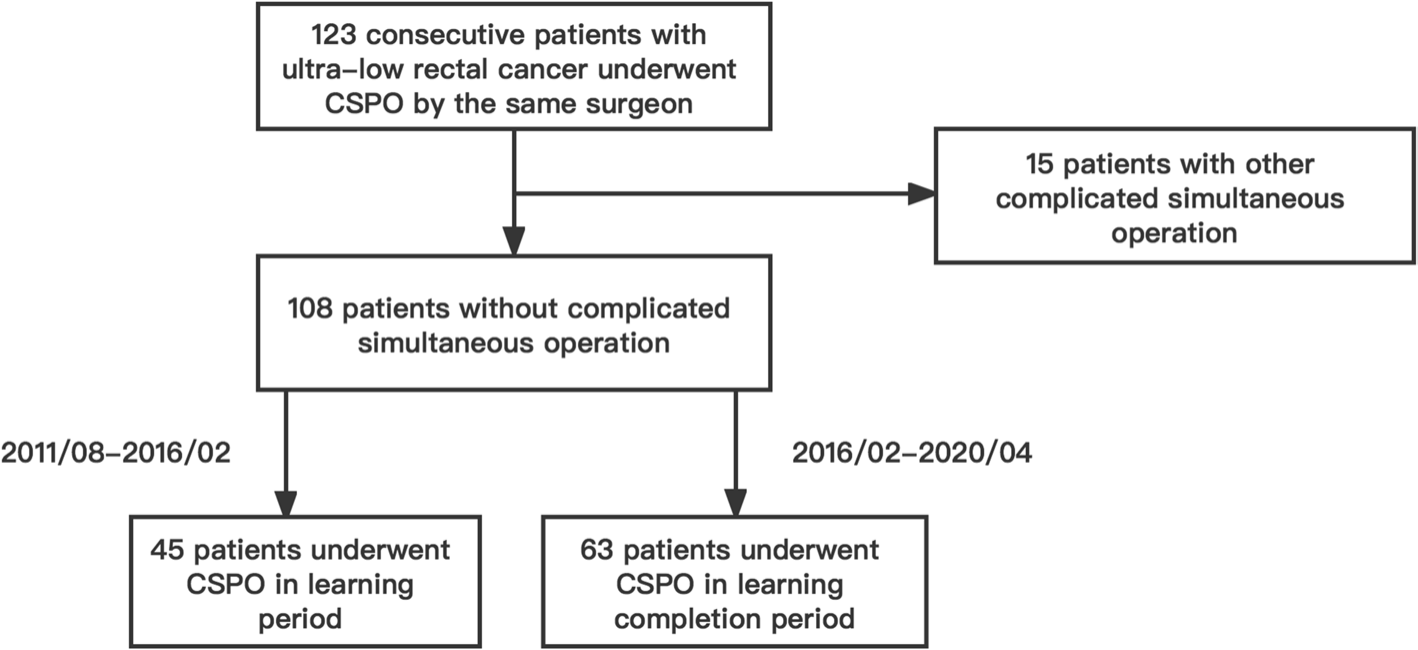
Learning curve flow chart

The CUSUM scatter plot of CSPO operation time (Fig. 2) was fitted with quadratic and cubic curves, and the equations were obtained: *Y* = 87.09 + 34.13X–0.33*X*^2^, *R*^2^ = 0.915; *Y* = −86.94 + 52.86*X*–0.76*X*^2^+ 2.62–3*X*^3^, *R*^2^ = 0.957. The goodness of fit of the cubic equation is higher, and the cutoff point value *X* = 45.4654. Then the CSPO learning curve can be divided into learning period (1–45 cases) and learning completion period (46–108 cases) (Fig. 3).

### Comparison of baseline characteristics of patients in different periods

There were no significant differences in preoperative clinical baseline data (gender, age, BMI, ASA classification, previous abdominal surgical history, neoadjuvant therapy, cT staging, cN staging, CEA, CA19-9); surgical and postoperative pathological data (the number of harvested lymph nodes, distance of tumor from anal verge, DRM, maximum diameter of mass, preoperative tumor gross type, the degree of tumor differentiation, TNM staging); and postoperative complications and survival data (postoperative complications, postoperative infection, classification of complications, and PFS3). However, the intraoperative blood loss in the learning period were significantly greater than those in the learning completion period, and the difference was statistically significant (*P* < 0.05). Besides, the flatus and defecation time, the liquid diet time, and the postoperative hospital stay in the learning period were significantly longer than those in the learning completion period, and the difference was statistically significant (*P* < 0.05). The median duration of follow-up in learning period was 76 (IQR, 55–108) months. The median duration of follow-up in learning completion period was 32 (IQR, 6–55) months. The higher OS3 in the learning completion period may be due to the fewer cases in the two periods and the shorter follow-up time in the learning completion period (Table 1).

### Univariate and multivariate analysis of influencing factors of CSPO operation time

Univariate and multivariate analysis showed that distance of tumor from anal verge (≥ 4 cm vs. < 4 cm, *P* = 0.039) and T stage (T3 vs. T1-2, *P* = 0.022) was independent risk factors for prolonged operation time of CSPO (Table 2, Table 3).

**Table 2:** Univariate analysis of influencing factors of CSPO operation time

	A vs. B			B vs. C		
	OR	95% CI	*P*	OR	95% CI	*P*
Age (》 65 years)	2.105	0.706~6.280	0.182	1.75	0.571~5.360	0.327
Gender (male)	0.727	0.280~1.887	0.513	1	0.375~2.664	1
BMI (》 25 Kg/m^2^)	1.203	0.431~3.356	0.724	1.203	0.431~3.356	0.724
Previous abdominal surgical history (yes)	0.357	0.098~1.300	0.118	2.653	0.294–23.922	0.385
Distance of tumor from anal verge (cm)						
2 ≦	1			1		
2 < *< 4	0.197	0.049~0.790	0.022		0.064–1.425	0.13
≧ 4	0.46	0.159~1.328	0.151		0.105–0.867	0.026
Maximum diameter of mass (cm)						
2 ≦	1			1		
2 < * < 4	1.591	0.395~6.407	0.514	1.909	0.563–6.477	0.3
≧ 4	0.625	0.181~2.155	0.457	1.455	0.454–4.655	0.528
ASA classification	2.57	0.812~8.132	0.108	1.123	0.353–3.567	0.845
Neoadjuvant therapy (yes)	4.6	1.4~15.117	0.012	1.6	0.610–4.139	0.339
CE (》 5 ng/ml)	1.395	0.440~4.424	0.572	1.395	0.440–4.424	0.572
CA19-9 (》 37 U/ml)	0.308	0.048~1.964	0.213	0.481	0.064–3.614	0.477
T stage						
T0	1			1		
T1	1.436	0.00~2.176	0.999	1.974	0–2.324	0.999
T2	1	0.260~3.845	1	2.2	0.54–8.957	0.27
T3	2.424	0.747~7.862	0.14	3.333	1.088–10.211	0.035
N stage						
N0	1			1		
N1	1.119	0.096~13.032	0.928	0	0–2.221	0.999
N2	0.615	0.034~7.452	0.615	0	0–2.324	0.999
TNM comprehensive staging						
0	1			1		
I	1.615	0~2.157	0.999	1.615	0–2.221	0.999
II	2.059	0.577~7.341	0.266	2.5	0.688–9.084	0.164
III	1.75	0.329~9.298	0.511	1	0.214–4.674	1

**Table 3:** Multivariate analysis of influencing factors of CSPO operation time

	A vs B			B vs C		
	OR	95% CI	*P*	OR	95% CI	*P*
Distance of tumor from anal verge (cm)						
2 ≦	1			1		
2 < * < 4	2.388	0.548–10.404	0.246	1.15	0.207–6.387	0.873
≧ 4	5.18	1.086–24.701	0.039	4.677	0.785–27.867	0.09
Neoadjuvant therapy (yes)	2.973	0.793–11.143	0.106	0.902	0.282–2.888	0.863
T stage						
T0	1			1		
T1	1.097	0–1.253	0.999	2.63	0–2.324	0.999
T2	0.81	0.175–3.759	0.788	1.599	0.317–8.068	0.57
T3	2.018	0.557–7.316	0.285	4.378	1.239–15.470	0.022

## Discussion

Although CSPO is also divided into transabdominal and transanal surgery, it is different from ISR [11] or ultralow Dixon. The first is to retain the internal anal sphincter and dentate line as much as possible. Then, the design of tumor resection line and conformal resection was carried out on the premise of the safety of tumor distal margin. In addition, the mechanical suture and manual suture of the excised conformal bowel are also different. These are not only the key reasons for the increased difficulty of CSPO but also the key points need to be overcome in the process of learning and training. Therefore, the definition of CSPO learning curve is crucial for beginners to formulate training plan, select appropriate cases, and set phased objectives. In addition, it also concerns the further promotion of CSPO.

Compared with previous studies on learning curve [20], this study explores CSPO learning curve through moving average, CUSUM, which are two more accurate statistical methods. It not only proves the existence of CSPO learning curve but also defines the cutoff point of learning curve in 45 cases. That is, beginners can pass the learning curve after completing 45 cases of CSPO. This is consistent with the 30–70 cases reported for learning curve in laparoscopic rectal cancer surgery [21, 22]. In addition, due to the need for conformal resection and anastomosis through the anus, the surgical approach of CSPO is similar to transanal total mesorectal excision (TaTME) and sphincter saving surgery. The results of CSPO learning curve are also consistent with 40–70 cases of TaTME [23, 24], and lower than 52 cases of robotic sphincter saving surgery [25], which also reflects the feasibility of CSPO learning and training.

According to the 45 cases of cutoff point of learning curve, the patients were divided into learning period and learning completion period. Subsequent statistical analysis proved that there was no significant difference between the two groups in curative resection (DRM, number of harvested lymph nodes), postoperative complications, and PFS3. This reflects the safety of learning period, and the surgical effect and prognosis will not be affected by surgical proficiency. Therefore, beginners can actively try this operation. In addition, it also proved that the learning completion period not only achieved a significant reduction in the operation time and intraoperative bleeding but also achieved a faster recovery of postoperative gut function and a shorter postoperative hospital stay. This is not solely related to the improvement of surgical proficiency but also related to the enhancement of the cooperation ability of the overall operation team, which also reflects the concept of fast-track surgery [26].

This study also further investigated the influencing factors of CSPO operation time, in order to use our experience in CSPO to help beginners choose the appropriate cases and get through the learning curve faster.

Many previous studies have proved that obesity is an important risk factor for prolonged operation time [27]. Especially for ultralow rectal cancer, obesity and narrow pelvis will increase the difficulty of free and anastomosis, thus increasing the operation time [28]. However, this study found that BMI was not an independent risk factor affecting the operation time of CSPO. On the one hand, this may be related to the bias between patient selection and enrollment in this study. That is, when doctors perform CSPO, the obesity degree is also a potential enrollment tendency. It can be seen that the body types of patients in this study are relatively moderate, and the BMI value is 23.0 ± 3.3kg/m^2^. On the other hand, it is related to the fact that CSPO does not require cutting and anastomosis in the abdominal cavity. That is, the anastomosis process of coloanal canal is carried out through the anus, which breaks through the limitation of the patient’s narrow pelvic space [29]. This reduces the impact of obesity on the operation time, which is also the advantage of CSPO.

At present, neoadjuvant therapy combined with TME and postoperative consolidation chemotherapy has become the standard treatment mode for advanced rectal cancer recommended by NCCN and ESMO guidelines. For ultralow rectal cancer, nCRT can not only reduce the tumor size and the difficulty of operation but also lower the tumor stage and achieve the criteria of anus preservation. However, many studies have shown that radiotherapy can lead to rectal wall fibrosis [30, 31]. This fibrosis will disturb the normal anatomical layer of perianal tissue, reduce the tactile feedback, increase intraoperative bleeding, prolong the operation time, and reduce the postoperative anal function [32, 33]. However, multivariate analysis in this study showed that nCRT was not an independent risk factor affecting the operation time. On the one hand, this is because the sphincter space is dissected through the anus under direct vision, which can facilitate hemostasis and clarity of anatomical layer, which is also the advantage of CSPO. On the other hand, many studies have shown that nCRT will cause microscopic morphological changes of internal anal sphincter [34, 35] but have little effect on its macroscopical morphology. Therefore, as long as we pay attention to the anatomical layer, the difficulty of operation will not increase too much.

In terms of the distance of tumor from anal verge, this study showed that the distance of tumor from anal verge ≥ 4 cm was an independent risk factor affecting the operation time of CSPO. This is different from the previous cognition that the closer the tumor is to the anal margin, that is, the lower the tumor location, the greater the difficulty of operation. There are several reasons. Firstly, the higher the tumor location, the more difficult it is to expose the limited space through anus under direct vision, which is easy to bleed and confuse the anatomical layer, increasing the difficulty. In addition, the patients included in this study were all with ultralow rectal cancer whose lower edge of the tumor is less than 2 cm from the dentate line. The distance of tumor from anal verge ≥ 4 cm means that the patient’s anal canal is longer, and the tumor is deeper under direct vision, which is also related to the patient’s obesity. These will increase the difficulty of surgery. This is a significant difference between CSPO and other rectal cancer surgery and also shows the advantage that CSPO can achieve anal preservation in a lower position.

In terms of tumor staging, all patients included in this study met the criteria of types II and III of Rullier classification [36]. It is required that the external anal sphincter of the patients is not invaded, so all the enrolled patients are T1-3 stage patients. Consistent with previous studies, multivariate analysis showed that T3 stage was an independent risk factor for CSPO operation time. On the one hand, the late tumor stage is often accompanied by nCRT for a long time, which has a certain impact on the operation. On the other hand, many previous studies have shown that the T stage of the tumor, that is, the depth of tumor invasion, is significantly and independently related to the local recurrence of the tumor [37]. In addition, late T stage is related to the distal invasion, lateral invasion, and lymph node metastasis. Therefore, for patients with late T stage, surgeons often need to clean lymph nodes and perform local operations more carefully, which will prolong operation time.

There are still some limitations in this study. First, this is a single-center nonrandom retrospective study, so there are some biases and deficiencies in the design itself. Second, this study mainly focuses on one surgeon’s experience, and the operative quantity is not particularly large. Considering the heterogeneity between surgeons, other surgeons may have different results in CSPO. In addition, the surgeon in this study has accumulated experience in more than 100 cases of laparoscopic surgery, so the CSPO learning curve may be longer for novice surgeons. Third, this study takes the operation time as the main index to measure the operator’s experience accumulation, but in fact, there are many other factors that may affect the operation time. In addition to the operation time, other indicators, such as the amount of bleeding, can also be used to reflect the operator’s experience. Fourth, the prolongation of liquid diet time and hospital stay in the learning period may be partly due to the selection bias of the researchers. It is because more conservative strategies are often adopted in the early stage of a new operation to ensure its safety. Fifth, the cutoff value of learning curve and the results of univariate and multivariate analysis need to be further proved in clinical practice. For these reasons, a prospective, multicenter, and multi-index randomized controlled study is needed to further confirm the conclusions of this study and explore the methods to shorten the learning curve.

## Conclusions

This study not only proved the existence of CSPO learning curve by moving average and CUSUM methods but also clarified further that surgeons with laparoscopic surgery experience need 45 cases of CSPO to complete the learning curve. In addition, further univariate and multivariate analysis showed that distance of tumor from anal verge (≥ 4 cm vs. < 4 cm, *P* = 0.039) and T stage (T3 vs. T1-2, *P* = 0.022) was independent risk factors for prolonging the operation time of CSPO. The experience of CSPO in our center is that the key steps are transabdominal laparoscopic operation and transanal operation. Therefore, it is suggested that beginners should strengthen their understanding of perianal anatomy and have some experience in laparoscopic surgery, perianal disease surgery, and manual coloanal anastomosis technology. Initially, it is recommended to select patients with benign or early malignant tumors (< T3) whose tumor is less than 4 cm from the anal verge and then further select more complex cases after about 45 cases of CSPO. In this way, beginners can reduce the operation time, accumulate experience, build self-confidence, and shorten the learning curve on the premise of safety.

## Data Availability

The datasets used and analyzed during the current study are available from the corresponding author on reasonable request.

## Abbreviations

CSPO: Conformal sphincter preservation operation
CUSUM: Cumulative sum control chart
nCRT: Neoadjuvant radiotherapy and chemotherapy
TME: Total mesorectal excision
NOTES: Natural orifice translumenal endoscopic surgery
CRM: Circumferential resection margin
ASA: American Society of Anesthesiologists
pCR: Pathological complete response
PFS3: Progression-free survival at 3 years
OS3: Overall survival at 3 years
ISR: Intersphincteric resection
DRM: Distal resection margin
TaTME: Transanal total mesorectal excision
NCCN: National Comprehensive Cancer Network
ESMO: European Society for Medical Oncology
